# Annotations

**Published:** 1889-10-26

**Authors:** 


### Annotations.

The War Office might do worse than issue a small commission to investigate the nursing arrangements at Netley Hospital. It is not difficult to believe that soldiers with little or no training in nursing in the civilian sense are ill adapted to the work of carefully tending patients dangerously ill, especially when suffering from a disease like typhoid fever. We should be very glad to hear the views of the officers who have been treated at Netley when suffering from typhoid, and also of the friends of those patients who have died there from this disease. Do the soldier nurses carefully tend such cases, and really nurse them as they would be nursed at tne London Fever Hospital, for instance, or are patients so affected left, at any rate, at times or at all, to attend to themselves? If any patient when dangerously ill has been so left, and has in consequence been forced to get out of bed, for example, a grave responsibility must rest upon those responsible for the administration of the hospital. It would be satisfactory to the public if a clear answer could be given to these questions, and the service papers might usefully ventilate the subject in the best interests of many a poor sufferer. We fail to see why the nursing of Netley Hospital should not at once be handed over entirely to a staff of certificated trained nurses under the control of a lady superintendent of nursing. That it is not so nursed at present is little to the credit of the country, and shows a very sad neglect of the officers and soldiers who seek relief within the walls of our chief military hospital.

**Nursing at Netley.**

The present is an age of self-elected experts on most subjects, who not infrequently make a noise in exact proportion to their ignorance of the questions they most affect. Even journalism is not free from midges of this description, and an amusing instance was supplied last week by "A Representative Organ," as it describes itself, for the fraternity in question like high-sounding phrases and big words with capital letters. This "organ" has taken matrons and nurses especially under its wing, although its lectures are frequently far from edifying to the real nursing experts, *i.e.*, to the workers and thinkers whom it essays to teach their business and duties. Recently it told its readers in the same article (1) that in most nurse-training schools the governing body has the entire power to select every officer, from the matron to the probationer, and after insisting that this was right and proper, it proceeded to declare (2) that the "choice of the workers must naturally be made by the individual most capable of rightly estimating their qualifications—*i.e.*, the matron." One paradox was not enough for "Mr. Editor," however, and so she generously contradicts herself again in the same article, being possibly carried away by the excitement of grinding the particular axe (matron) she is always striving to bring into contempt. So she declares that this particular matron's committee (1) only sits upon sufferance, as its members are entirely ignorant of what transpires in the institution, and (2) that the same committee displayed ample grasp of its responsibilities on a recent occasion, and acted up to them in a manner quite satisfactory to the writer. Now, as a matter of fact, with the exception of the hospital to which "Mr. Editor" is supposed to have belonged, where she formerly ruled with a rod of iron, and where the rules provide that the matron shall hire and dismiss at her pleasure, the best hospital matrons only have power to select and suspend the staff, appointment and dismissal being subject to the approval of the committee. Responsibility is a very rich dish, and matrons, like other people in authority, soon find that it can be served out to all who care to partake of it, and still there will be more than enough left for themselves to bear. Even the most elementary knowledge of hospital management would have supplied "Mr. Editor" with these elementary facts, and so spared her any annoyance resulting from the amusement, which this particular tune on the "representative (but faulty) organ," has created in hospital circles.

**The Matron's Powers.**

The appearance of the first grey hair, like the arrival of a new baby, is certainly an event, and particularly to womankind. If Nature were disposed to be extremely considerate, she might permit us to grow old without blazoning the fact abroad to all the world by means of whitening whiskers and hair powdered without the aid of the powder-box. Men and women have many controversies with Nature about their height, or their bandy legs, or their flat noses, or what not; but perhaps the one standing quarrel that knows no signs of truce is that caused by her unflinching determination to make us turn greyer and greyer whilst we are still young—quite young, "you know." Many have been the attempts of sympathetic professors of the hairdressing art to plant themselves like Horatius on the bridge and to fight the remorseless enemy. But the success has been more than doubtful. The writer well remembers a broad giant of a man who used to frequent the fashionable promenade of a university town, whose hair was of midnight blackness, and of more than midnight gloom. It was simply hideous. He can call to mind also the case of another man of about forty, whose beard turned grey prematurely, and whose wife insisted upon trying a decoction which was warranted "not a dye." "A dye," however, it proved to be, and that of a most charmingly mongrel type, a kind of mixture between a faint magenta and a lively pink. The rejuvenated beard was a marvel of the dyers' art; and if the colour had been permanent, which happily it was not, there is no doubt that Barnum would by this time have insisted upon purchasing both the magenta beard and its owner at any cost. But a Regent-street firm are now declaring that the problem has been solved at last. Their new product is "not a dye," but "a stain." Mark the fine classical distinction. The firm have evidently never discussed the baptismal controversy, or they would know that in New Testament Greek the one word "baptizo" may mean "to stain," or "to dye," "to dip," or "to sprinkle," "to plunge," or "to pour," and several other things. But the new "stain" is to work wonders. Grey hair is to be converted into "black brown," or even into "auburn." Or, if the owner chooses to submit himself to "experienced assistants," his grey locks may be turned into the "lightest flaxen," or the "darkest brown." Happy man and fortunate woman! What fee in solid gold or crisp bank notes can repay this most meritorious firm and their benevolent "experienced assistants"? "Ladies' thin partings"—an unpardonable suggestion, for ladies never have thin partings; but if by some malign influence and conflux of the heavenly bodies any lady within the four corners of the earth should have a "thin parting," an "invisible foundation" can be at once supplied, and on it can be raised the noblest superstructure of beautiful and youthful "black" or "auburn" to match. "Sigh no more, ladies: ladies, sigh no more." The days of deceivers are over. The "Mystax-stimulo" will work all the wonders it promises, as surely as "Mesopotamia" was potent in Whitfield's mouth to produce the immediate reformation of a washerwoman's morals. Or if "Mystax-stimulo" should fail, then recourse may be had to "Trichobamma," or to "Eau Persane," or to "Neroli Elixir," or "Ruber Aurum," or "Liquid Kohol Egyptian," or anything else you please. Such, then, is the pass to which modern civilisation has brought us—that a whole column of stuff of this kind may be advertised at an immense cost in a morning paper, and that there are plenty of fools in England to make the advertisement profitable. Is it possible? It must be both possible and true. There is no proof so convincing as the proof that is demonstrated by the expenditure of hard cash. Let us hope that in the millenium all the women will be bald and the men will shave. We shall then, at least, be saved from the gigantic impudence of hair-dyes.

